# Characterization of basigin monoclonal antibodies for receptor-mediated drug delivery to the brain

**DOI:** 10.1038/s41598-020-71286-2

**Published:** 2020-09-03

**Authors:** Sarah Christine Christensen, Berit Olsen Krogh, Allan Jensen, Christian Brix Folsted Andersen, Søren Christensen, Morten Schallburg Nielsen

**Affiliations:** 1 Department of Biomedicine, Aarhus University, Høegh-Guldbergsgade 10, Building 1116, 8000 Aarhus C, Denmark; 2 Department of Biotherapeutic Discovery, H. Lundbeck A/S, Copenhagen, Denmark

**Keywords:** Fluorescence imaging, Biologics, Drug delivery

## Abstract

The brain uptake of biotherapeutics for brain diseases is hindered by the blood–brain barrier (BBB). The BBB selectively regulates the transport of large molecules into the brain and thereby maintains brain homeostasis. Receptor-mediated transcytosis (RMT) is one mechanism to deliver essential proteins into the brain parenchyma. Receptors expressed in the brain endothelial cells have been explored to ferry therapeutic antibodies across the BBB in bifunctional antibody formats. In this study, we generated and characterized monoclonal antibodies (mAbs) binding to the basigin receptor, which recently has been proposed as a target for RMT across the BBB. Antibody binding properties such as affinity have been demonstrated to be important factors for transcytosis capability and efficiency. Nevertheless, studies of basigin mAb properties' effect on RMT are limited. Here we characterize different basigin mAbs for their ability to associate with and subsequently internalize human brain endothelial cells. The mAbs were profiled to determine whether receptor binding epitope and affinity affected receptor-mediated uptake efficiency. By competitive epitope binning studies, basigin mAbs were categorized into five epitope bins. mAbs from three of the epitope bins demonstrated properties required for RMT candidates judged by binding characteristics and their superior level of internalization in human brain endothelial cells.

## Introduction

Antibodies as therapeutic modalities are highly attractive due to their target specificity, long serum half-life, mechanism of actions, and limited off-target effects compared to small molecules and peptides. During the past decades, huge efforts have been made to optimize the delivery of biotherapeutics across the blood–brain barrier (BBB), which is a major limiting factor for successful antibody treatment of central nervous system-associated disorders. Systemic administration of antibodies results in low brain exposure of < 1% of the injected dose^1,2^. The low permeability is due to the efficient tight junctions of the brain endothelial cells that form the front line of the BBB, preventing paracellular diffusion of large molecules into the brain. Most of the research effort has been focusing on enhancing transcellular transport as a non-invasive option. The most promising brain delivery strategy of antibodies relies on utilizing endogenous transport mechanisms, such as receptor-mediated transcytosis (RMT), by receptors present at the surface of brain endothelial cells to bypass the BBB. The transferrin receptor 1 (TfR1) has been explored extensively as a target for RMT, and in 1987 Fishman et al.^3^ succeeded in enhancing brain exposure of antibodies specific for the TfR1. Since then, there has been an increasing interest in developing receptor-mediated drug delivery platforms to enhance brain uptake of central nervous system biotherapeutics. Targeting the TfR1 using antibodies with retained Fc effector led to safety liabilities such as a reduction in circulating reticulocytes, microglial activation, and astrogliosis^4,5^. Although risk mitigation strategies are under development^6,7^, alternative drug delivery targets at the BBB are still explored.

Zuchero et al.^1^ investigated brain uptake mediated by other high abundant receptors and transporters in brain endothelial cells. One of the investigated receptors was the basigin receptor (also known as CD147, neurothelin, 5A11, EMMPRIN, gp42, HT7, OX47, CE9, M6, and HAb18G). They reported similar brain accumulation of anti-basigin and anti-TfR1 antibodies in a mouse model. In addition to enhanced delivery into the brain, the basigin receptor is also highly attractive due to its low expression in neurons^8^. This is favorable since the mode of action of many brain therapeutic antibodies requires an extracellular target engagement (e.g., amyloid-β, α-synuclein, and tau aggregates), where uptake into neurons is unwarranted. The basigin receptor was first identified in mouse B16 melanoma cells and is part of the immunoglobin (Ig) superfamily^9,10^. By comparing mouse brain endothelial cells to liver and lung endothelial cells, basigin is 14 fold enriched in brain endothelial cells based on microarray expression analysis^11^. Moreover, basigin expression was found to be upregulated under ischemic conditions as well as in multiple sclerosis and Alzheimer's disease patients^12–15^. Basigin is a highly glycosylated type 1 transmembrane protein and a co-receptor for the lactate transporter, monocarboxylate transporter 1, present at the BBB^16^. Basigin has four isoforms where the most abundant is the isoform 2 with two extracellular Ig-like domains with intra-domain disulfide bonds^17^. Basigin has a highly conserved single transmembrane region and a short cytoplasmic tail^18,19^. The extracellular domain contains three N-glycosylation sites^20^. Basigin exists in different glycosylated forms – highly glycosylated and lowly glycosylated around 50 and 38 kDa, respectively^21–23^. The glycosylation has been suggested to be essential for basigin's function and localization to the plasma membrane^22,24^. The subcellular sorting and transcytosis of the basigin receptor in brain endothelial cells have not been well-explored, and most published data is based on trafficking in MDCK cells or non-polarized HeLa cells. After thoroughly studying the TfR1 as a transcytosis-shuttle, it appears likely that for an antibody targeting the basigin receptor via RMT, receptor binding characteristics, such as affinity, valency, pH-dependency, and epitope, would also be crucial for improved brain exposure. It has been reported extensively, that sorting of the antibodies to lysosomes affects the BBB-crossing ability of the antibodies. For TfR1 antibodies, lysosomal localization was suggested to be affected by binding affinity^25,26^, bivalent target interaction (i.e., avidity binding)^27^, and pH-dependency of receptor binding^28^. Hence binding epitope, affinity, and the functional impact of antibody binding may well influence the behavior of the target receptor. Consequently, receptor internalization, subcellular trafficking, and signaling might be affected differently across a diverse panel of antibodies.

In this study, we isolated a number of mouse monoclonal antibodies (mAbs) against the basigin receptor to select a set of candidates for basigin-mediated delivery across the BBB. The first step in our characterization was to define bins of antibodies with overlapping basigin epitopes. Subsequently, representatives from the different bins were subjected to further characterization of affinity, cellular binding and localization, and the ability to internalize. Here we showed a comprehensive characterization of a large panel of basigin antibodies and selection of quality lead candidates potentially to be used in the engineering of BBB crossing antibody constructs.

## Results

### Generation of novel basigin monoclonal antibodies

Anti-basigin mAbs were generated by standard hybridoma technology. Mice were immunized with recombinant forms of human basigin, corresponding to the extracellular domain of the receptor (basigin-ECD). Hybridomas were screened by enzyme-linked immunosorbent assay (ELISA) for binding to human basigin-ECD and cross-reactivity to rat, mouse, and porcine basigin-ECD. Thirty-two hybridomas with high titers were selected for V-gene recovery and sequencing. A few of the hybridomas were non-clonal, yielding multiple light chain (LC) and heavy chain (HC) sequences. By including all HC/LC combinations from clonal and non-clonal hybridomas, a final set of 54 mAbs were produced recombinantly as human IgG1/κ (G1m3) chimeras for further characterization (see methods in supplementary materials).

### Epitope binning of basigin monoclonal antibodies

**Figure 1:**
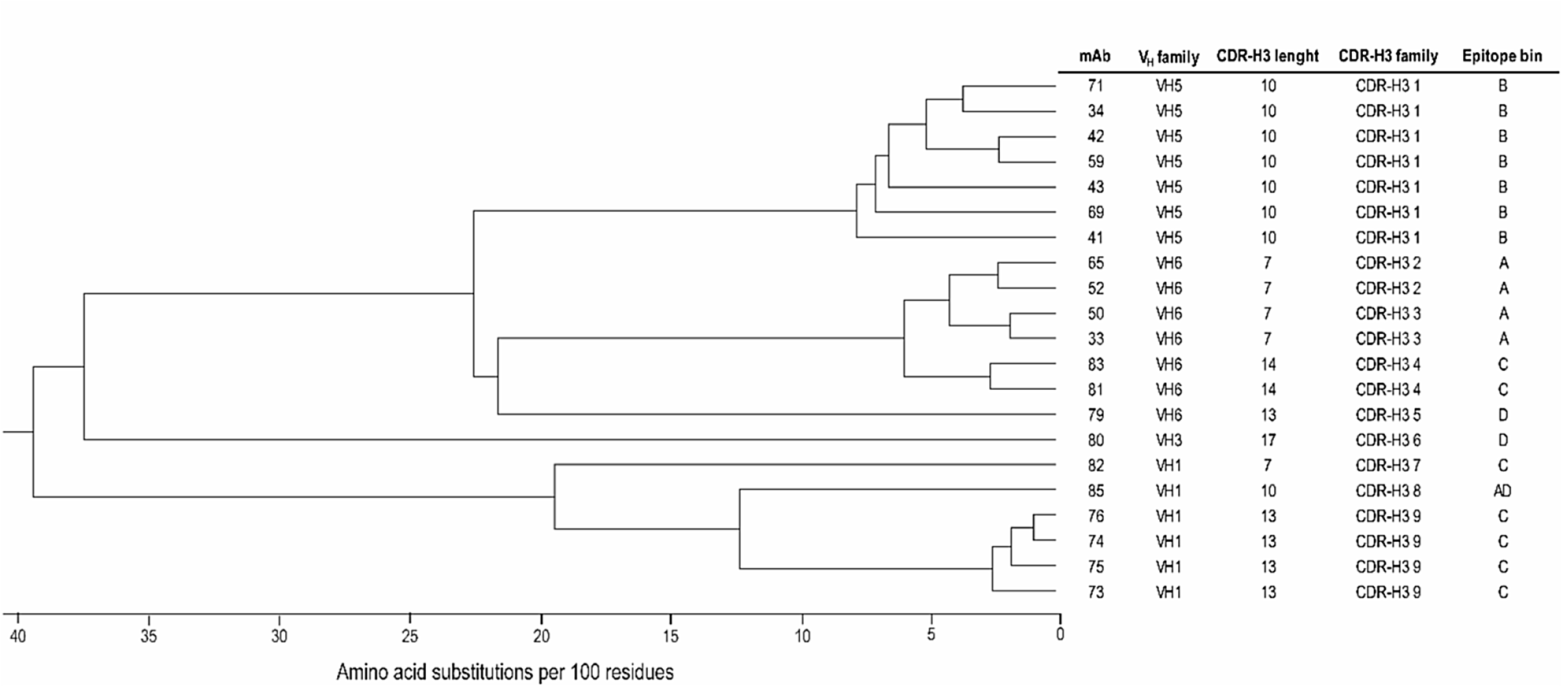
Phylogram of 21 basigin monoclonal antibodies (mAbs). The phylogram was produced with Multiple Sequence Comparison by Log-Expectation (MUSCLE) alignment using heavy chain variable domain (V_H_) sequences with the Unweighted Pair Group Method with Arithmetic Mean (UPGMA) algorithm, Jukes-Cantor distance measure, and 10,000 Bootstrap replicates. The heavy chain CDR3 (CDR-H3) families are determined based on an identical length and more than 80% amino acid sequence identity. CDRs and mouse V_H_ germline families were annotated according to IMGT. V_H_ family: germline family of the heavy chain variable domain.

**Figure 2:**
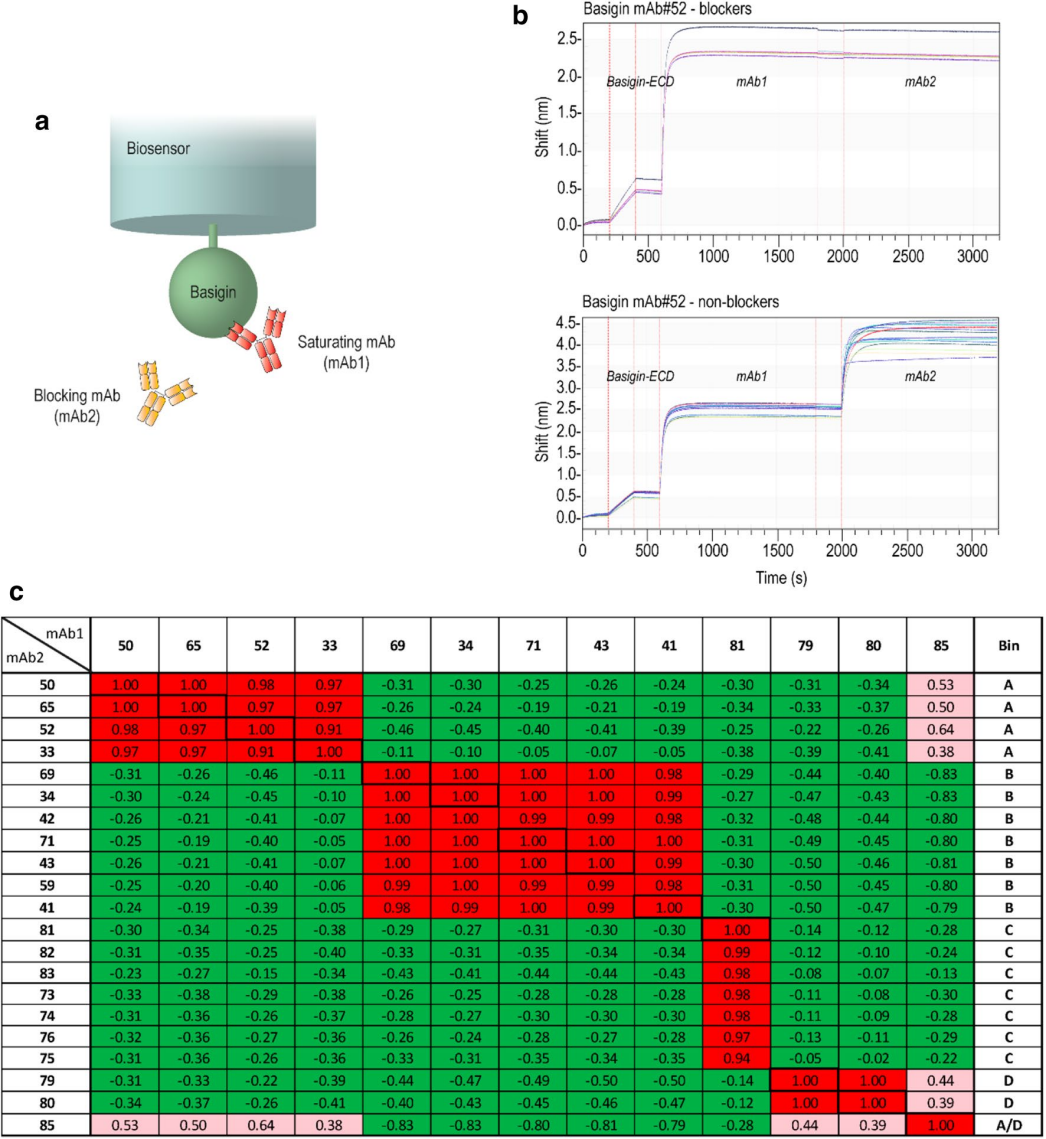
Epitope binning assay of the basigin monoclonal antibodies (mAbs). (**a**) Schematic representation of the epitope binning setup run in-tandem with basigin-ECD immobilized onto streptavidin biosensor followed by binding of the saturating mAb (mAb1) and subsequent the blocking mAb (mAb2). (**b**) Examples of sensorgrams with basigin mAb#52 as saturating mAb and blocking and non-blocking antibody pairs. (**c)** Matrix with Pearson correlation coefficients with the saturating mAbs (mAb1) listed in columns, and the blocking mAbs (mAb2) in rows. The highest correlations are marked in red, defined as the Pearson's correlation coefficient above 0.9, and the weak correlation in light red as between 0 and 0.9. Negative Pearson's correlations marked in green, indicating no cross-blocking between antibody pairs. Antibody self-binding pairs are boxed with bold lines. The assigned epitope bins are listed in the rightmost column.

Initially, the 54 basigin mAbs were tested for binding to immobilized human basigin-ECD using biolayer interferometry (BLI), which reduced the number of mAbs to 21 positive binders. Alignment of heavy chain variable domain (V_H_) sequences was performed to explore the diversity of the basigin antibody panel. The alignment was used to generate a phylogram for the V_H_ sequences (Fig. 1). The individually V_H_ complementarity-determining region 3 (CDR-H3) families, defined by an identical length and more than 80% amino acid sequences identity, should identify antibodies from the same V-D-J recombination lineage. The CDRs were designated according to the IMGT annotation scheme. LC sequences were not included in the phylogenetic tree. However, the LC sequences were almost identical within the CDR-H3 families. The 21 aligned mAbs were representing nine different CDR-H3 families, subsequently grouped into five epitope bins. In-tandem BLI was used to identify the epitope bins (Fig. 2). Antibodies from the same CDR-H3 family are expected to bind the same epitopes, i.e., belong to the same bin. Here antibodies that compete for antigen binding in a cross-blocking matrix are assigned to the same epitope bin. The biosensors were loaded with recombinant basigin-ECD and sequentially introduced to a saturating mAb (mAb1) and a blocking mAb (mAb2) (Fig. 2a). Examples of blocking profiles from a blocking and a non-blocking antibody pair are depicted in Fig. 2b. The epitope binning data was summarized in the matrix showing the Pearson correlation coefficients between the antibodies blocking profiles, which were calculated based on normalized binning data (Fig. 2c). Antibodies with high positive Pearson correlation coefficient (above 0.9) were placed into the same bin (marked in red). The mAbs in light red indicate a positive Pearson correlation coefficient between 0 and 0.9. Green indicates antibody pairs with non-overlapping epitope regions. All antibodies were run in a self-blocking setup as controls (boxed in the matrix with bold lines). The binning experiment resulted in the mapping of five distinct epitope bins (A, B, C, D, and AD) (rightmost column in Fig. 2c). Basigin mAb #85 showed a unique profile with cross-blocking of antibodies in both bin A and D, indicating that bin A and D epitopes are either in structural proximity or neighboring peptide stretches. Basigin mAbs with a baseline drift after loading above 5% of total binding were only used as the blocking antibody (mAb2) to limit the chance for bin misinterpretation. Two bin B representatives were excluded as saturating mAb based on their fast off-rates (#42 and #59). However, basigin mAb#42 and #59 were in the same CDR-H3 family as the other bin B mAbs, indicating that they were correctly assigned to bin B despite its lack of verification in both assay directions. Similarly, only one (#81) of the seven bin C mAbs (#81, #82, #83, #73, #74, #76, and #75) could be confirmed in both assay orientations. As expected, antibodies from the same CDR-H3 families were mapped to the same epitope bind in the BLI assay (last column in Fig. 1). Overall, the alignment showed the diversity of the antibody panel with nine different CDR-H3 families grouped into five different epitope bins by the BLI assay. Bin A is represented by two CDR-H3 families, whereas bin B is comprised of a single CDR-H3 family. Bin C covers three CDR-H3 families, and bind D is represented by two antibodies from different CDR-H3 families. As could be expected, the single candidate representing bin AD belongs to a unique antibody lineage in the panel.

### Affinity determination by surface plasmon resonance

**Figure 3:**
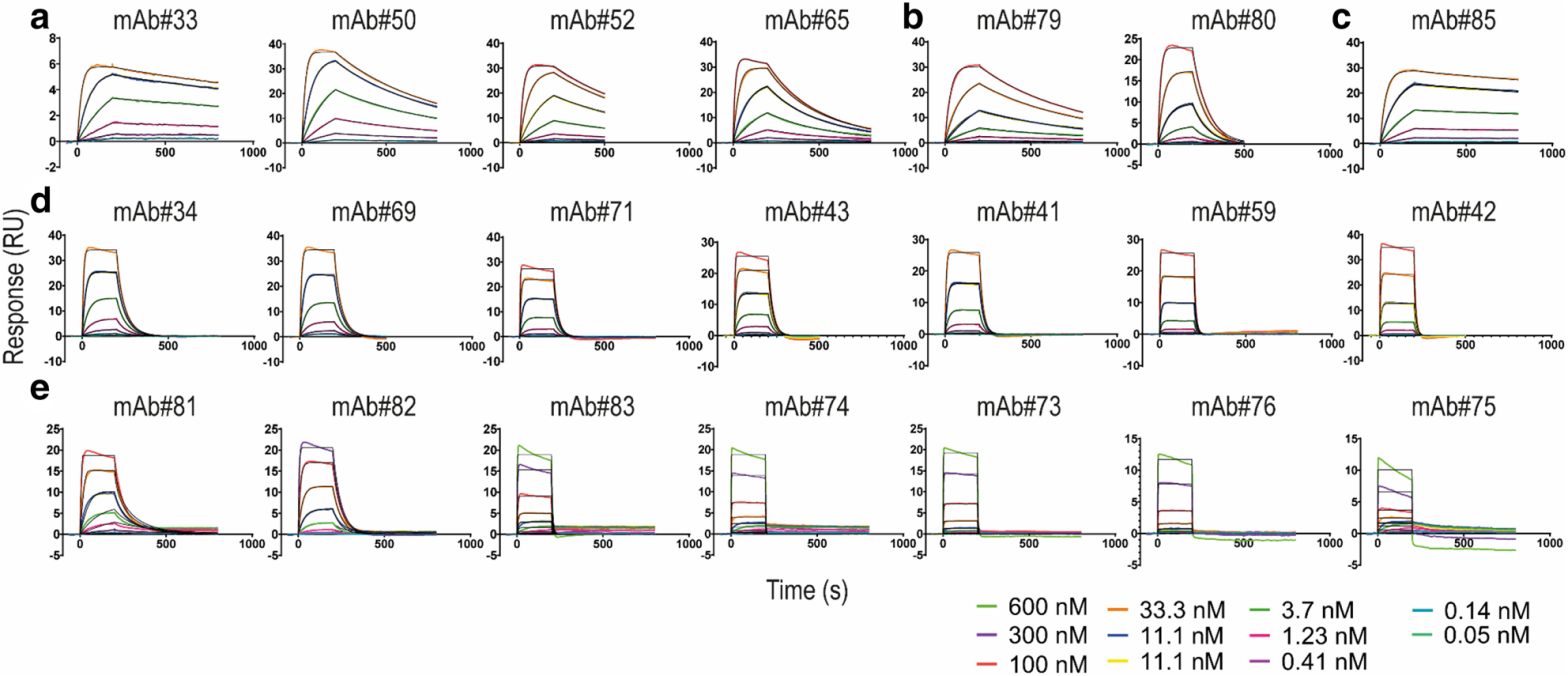
Surface plasmon resonance sensorgrams representing the binding of basigin extracellular domain to captured anti-basigin monoclonal antibodies. The fitted curves used for equilibrium dissociation constant (K_D_) calculations were obtained using the Langmuir 1:1 binding model in the Biacore S200 evaluation software and plotted in GraphPad Prism (colored in black). (**a**) bin A, (**b**) bin D, (**c**) bin AD, (**d**) bin B, and (**e**) bin C. *mAb* monoclonal antibody, *RU* response unit.

The 21 candidates identified in the binning experiments as positive binders were selected for kinetic profiling of their binding to recombinant basigin-ECD by surface plasmon resonance (SPR) (Fig. 3). A pattern between epitope bin and target affinity was observed by comparing the binding kinetics measured by SPR (Table 1). Bin A mAbs have the highest affinities, followed by bin B, whereas most of bin C mAbs were low in affinities. Basigin mAb#85 in bin AD showed strong binding with a K_D_ of 0.4 nM together with bin A antibodies that all have a high affinity in the low nanomolar range (0.3–4 nM). The basigin antibodies in bin B covered K_D_ values ranging from 7 to 40 nM. The bin B mAbs originate from the same CDR-H3 family, but the CDR-H3 sequences did not contribute to the differences in affinity observed within epitope bin B. Bin C mAb#81 and #82 had binding affinities ranging from K_D_ values of 10 to 20 nM, whereas mAb#73–76 and #83 had poor affinities with K_D_ values above > 100 nM. The K_D_ values of antibodies in bin D were calculated as 6 nM (mAb#79) and 20 nM (mAb#80). A slower dissociation of mAb#79 caused this difference. All bin A mAbs, mAb#85 (bin AD), and mAb#79 (bin D) showed fast association rates and slow dissociation rates. The dissociation rates were faster for bin B but showed similar association rates as the other epitope bins. Some of the kinetic constants were difficult to determine due to a poor data fit (Chi2 values in the last column in Table 1). The candidate set was dominated by antibodies with fast association kinetics, which posed a general challenge for proper kinetics analysis. The sensorgrams for basigin mAb#73–76 illustrated the combined fast association and dissociation rates observed for these antibodies, which made determining proper kinetic constants technically difficult (Fig. 3e). The fast on-rate kinetics observed for several candidates may contribute to the unspecific binding phase observed as an association "hump" in the sensorgrams (e.g., mAb#83 and mAb#73–76) and resulting in a corresponding negative response in the dissociation phase (Fig. 3e). The effect was particularly pronounced for mAb#75. For candidates with K_D_ values higher than 40 nM, ligand saturation was not reached as expected in the analyte titration from 0–600 nM. The binding stoichiometry, when calculated based on the observed maximal response (R_max_) and the theoretical R_max_ for a 2-binding site ligand, was 0.7 or less for the higher K_D_ antibodies.

**Table 1:** Surface plasmon resonance kinetic rate constants of basigin monoclonal antibodies.

mAb	Theoretical R_max_ (RU)	Measured R_max_ (RU)	Stoichiometry	k_a_ (1/Ms)	k_d_ (1/s)	Kinetic K_D_ (nM)	Chi^2^ (RU^2^)	Bin
85	32	32	1.0	9 × 10^5^	4 × 10^−4^	0.4	0.002	AD
33	6	6	1.0	2 × 10^6^	6 × 10^−4^	0.3	0.002	A
50	44	42	1.0	2 × 10^6^	2 × 10^−3^	1	0.025	A
52	38	34	0.9	6 × 10^5^	2 × 10^−3^	3	0.015	A
65	36	36	1.0	1 × 10^6^	4 × 10^−3^	4	0.055	A
34	44	43	1.0	5 × 10^6^	3 × 10^−2^	7	0.033	B
69	50	41	0.8	4 × 10^6^	4 × 10^−2^	9	0.122	B
71	32	32	1.0	4 × 10^6^	5 × 10^−2^	10	0.123	B
43	34	27	0.8	3 × 10^6^	5 × 10^−2^	20	0.240	B
41	42	37	0.9	4 × 10^6^	7 × 10^−2^	20	0.065	B
59	36	32	0.9	5 × 10^6^	2 × 10^−1^	30	0.115	B
42	52	34	0.7	4 × 10^6^	2 × 10^−1^	40	0.132	B
81	26	22	0.8	3 × 10^6^	3 × 10^−2^	10	0.091	C
82	32	22	0.7	1 × 10^6^	4 × 10^−2^	20	0.065	C
83	32	22	0.7	6 × 10^6^	1 × 10^0^	> 100	0.601	C
74	40	21	0.5	2 × 10^6^	5 × 10^−1^	> 100	0.966	C
73	38	20	0.5	9 × 10^5^	3 × 10^−1^	> 100	0.118	C
76	32	13	0.4	1 × 10^6^	7 × 10^−1^	> 100	0.074	C
75	54	12	0.2	4 × 10^6^	1 × 10^0^	> 100	0.652	C
79	38	35	0.9	3 × 10^5^	2 × 10^−3^	6	0.096	D
80	36	27	0.8	6 × 10^5^	1 × 10^−2^	20	0.047	D

*mAb* monoclonal basigin antibody, *R*_*max*_ Analyte binding capacity (2x), *k*_*a*_ Association rate constant, *k*_*d*_ dissociation rate constant, *Kinetic K*_*D*_ Kinetic equilibrium dissociation constant, *RU* response unit, *Bin* epitope bin determined by epitope binning experiment.

The constants are derived from the sensorgrams in Fig. 3 using the Langmuir 1:1 binding model fitted locally with the Biacore S200 and T200 evaluation software. The K_D_ values are average of two independent experiments with a standard deviation below 2 nM except mAb#82 with a standard deviation of 7 nM.

### Screening of cell association to human brain endothelial cells using flow cytometry

**Figure 4:**
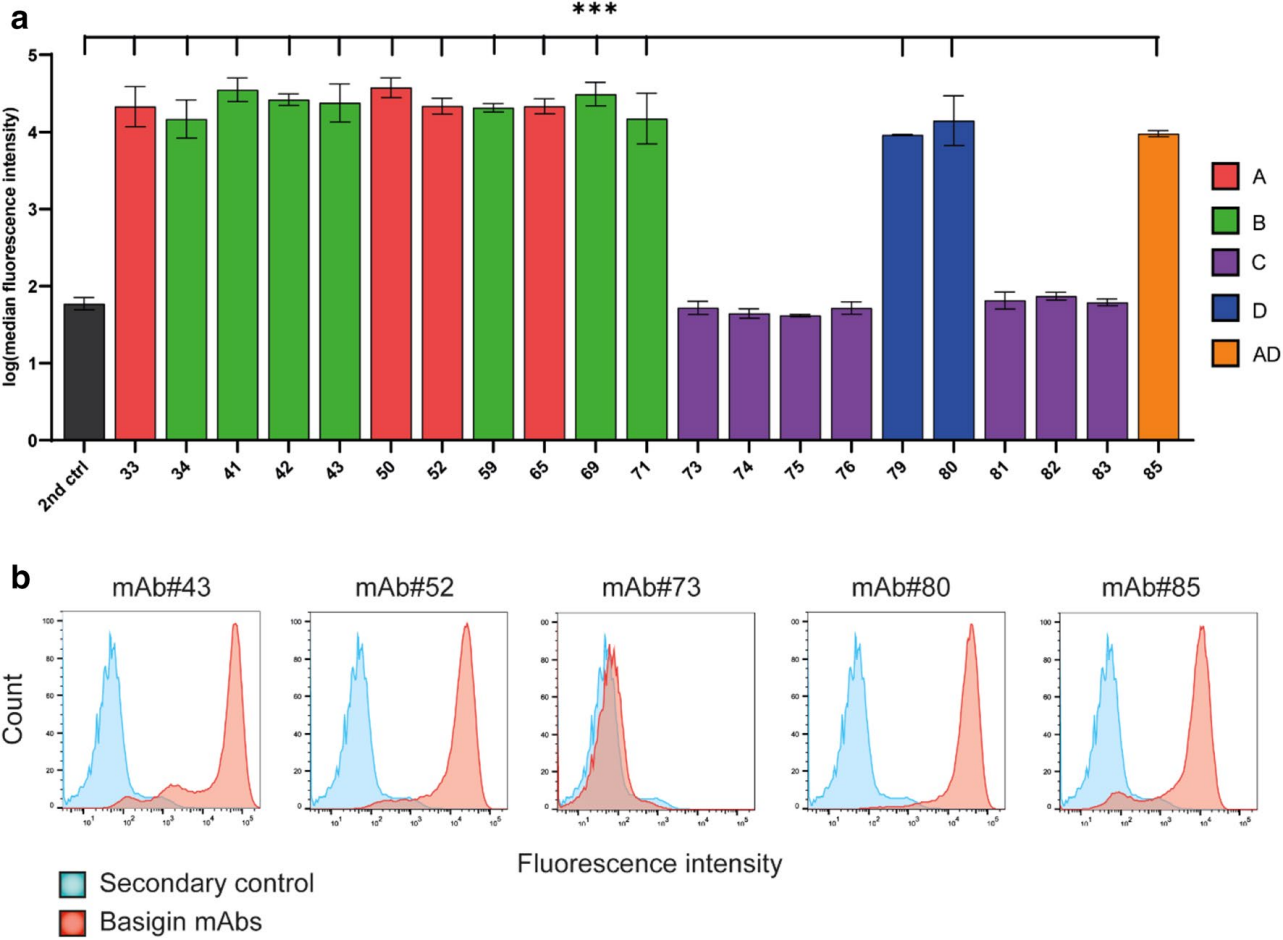
Basigin monoclonal antibody (mAb) association with hCMEC/D3 cells analyzed by flow cytometry. (**a**) The data was log-transformed and plotted with ± standard error of the mean (SEM). Multiple comparisons of hCMEC/D3 cell-association with the different basigin mAbs with controls cells stained only with the secondary antibody (2nd ctrl) were performed by one-way ANOVA with Dunnett's multiple comparison post hoc test. ***p < 0.001. (**b**) Selected histograms with the secondary control in blue and basigin mAbs in red. See also supplementary Figures S1 and S2 for gating strategy and the remaining flow cytometry histograms.

Since the BLI and SPR assays were done with a recombinant basigin-ECD, it was also important to screen all the basigin mAbs for binding to the cell surface-expressed basigin receptor. The evaluation of cell association was by flow cytometry using the human brain endothelial cell line, hCMEC/D3, which has endogenous expression of the basigin receptor. All the bin A, B, D, and AD mAbs were positive for binding the hCMEC/D3 cells with up to a 600-fold change in median fluorescence intensity compared to controls cells (2nd ctrl, stained only with the secondary antibody) (p-value < 0.001) (Fig. 4a). Histograms of selected mAbs representing each epitope bin visualize the shift in populations based on binding of the anti-basigin mAb to the receptor on hCMEC/D3 cells compared to control cells (Fig. 4b). The remaining flow cytometry histograms and the gating strategy can be inspected in supplementary Figure S1 and S2. No gating of negative and positive populations was applied due to the clean division of positive and negative cell populations. None of the bin C mAbs was capable of binding the native basigin receptor, as shown by the complete lack of a positive cell population and overlap with negative control cells (mAb#73 in Fig. 4b). Based on the kinetic profiling and cell association, the lowest affinity basigin mAbs (K_D_ values > 100 nM) were excluded from further characterization reducing the number evaluated from 21 to 16 mAbs.

### Immunofluorescence staining of hCMEC/D3 cells with different basigin monoclonal antibodies

**Figure 5:**
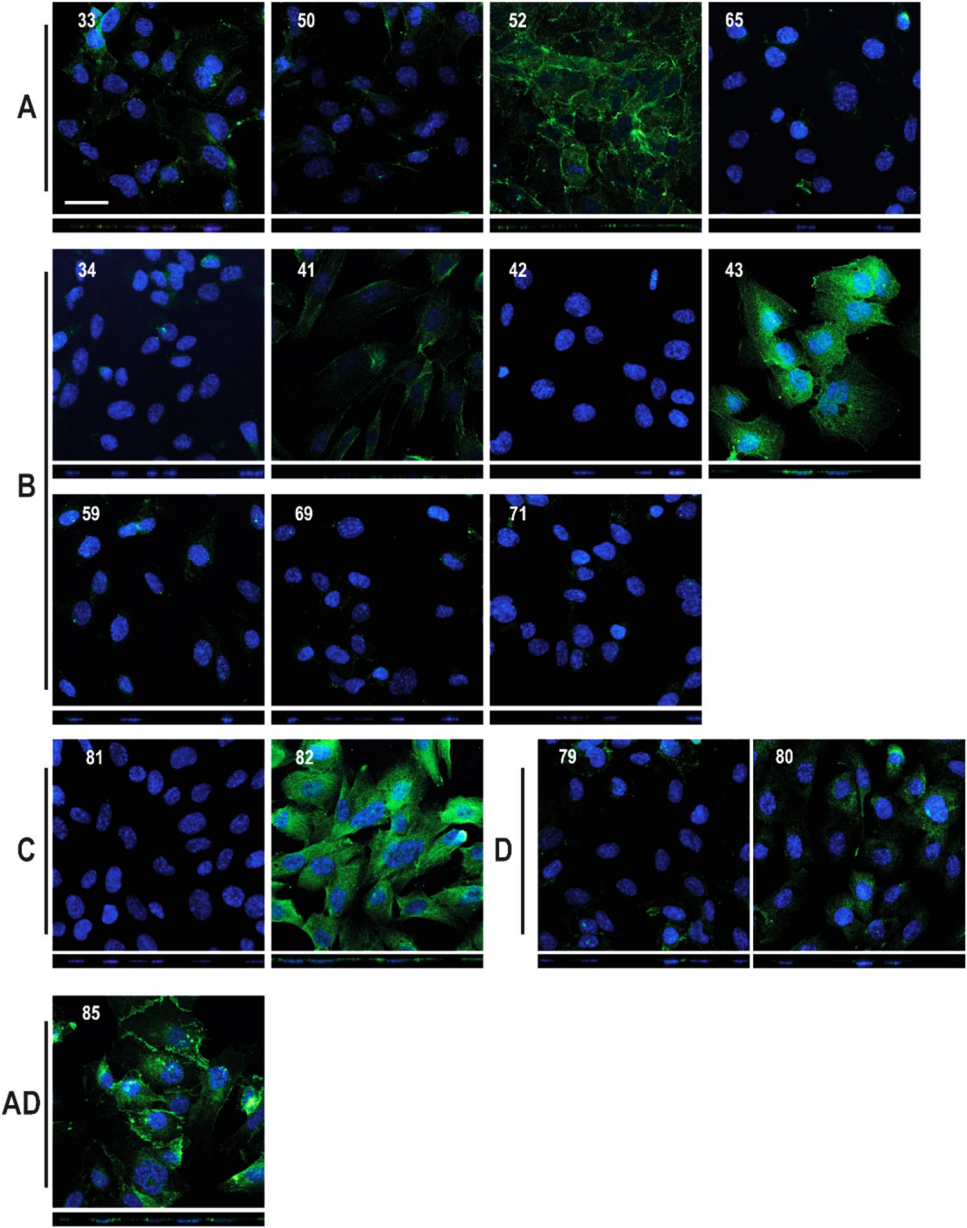
Immunofluorescence staining of basigin in hCMEC/D3 cells with selected basigin monoclonal antibodies. Representative microscopic images of basigin (green) in hCMEC/D3 cells. Nuclei visualized by Hoechst (blue). The pictures are the maximum projection of the z-stack, and the XZ projection is below the pictures. Scale bar 30 µm. The pictures are sorted depending on their epitope bin, as indicated to the left. *indicates that the pictures were processed differently compared to the rest due to avoid over-exposed images.

Sixteen representative mAbs from the different epitope bins were further characterized by immunofluorescence for their ability to interact with the basigin receptor in fixed hCMEC/D3 cells (Fig. 5). Bin A basigin mAbs generally bind the hCMEC/D3 cells with mAb#52 showing the strongest signal. Bin B antibodies yielded both strong, weak, and no binding signal on fixed hCMEC/D3 cells. Surprisingly, bin B mAb#69, #71, #42, and #59 were among the antibodies showing weak or no binding to the fixed cells even though these candidates showed strong binding to the hCMEC/D3 cells by flow cytometry analysis (Fig. 4). For bin C, where none of the antibodies associated with the living cells, mAb#82 showed differentiated, robust signal on fixed hCMEC/D3 cells. Bin D mAb#80 bound strongly to the cells, whereas mAb#79 only resulted in weak staining. The immunofluorescence staining of fixed cells can be used for examination of the subcellular binding pattern of the antibodies. Immunofluorescence staining with mAb#80 (bin D) seemed to be detecting a perinuclear localization of the basigin receptor (XZ projection pictures in Fig. 5). In contrast, bin A mAb#52 seemed to detect basigin receptors closer to the cell surface. Basigin mAb#41 (bin B) behaved differently compared to the rest of the antibodies displaying an actin-like staining pattern. In summary, the immunofluorescence staining revealed that some basigin mAbs were more influenced by fixation of the epitope than others and that different sub-populations of the receptor with different local concentrations and epitope representation may exist.

### Internalization of selected basigin monoclonal antibodies

**Figure 6:**
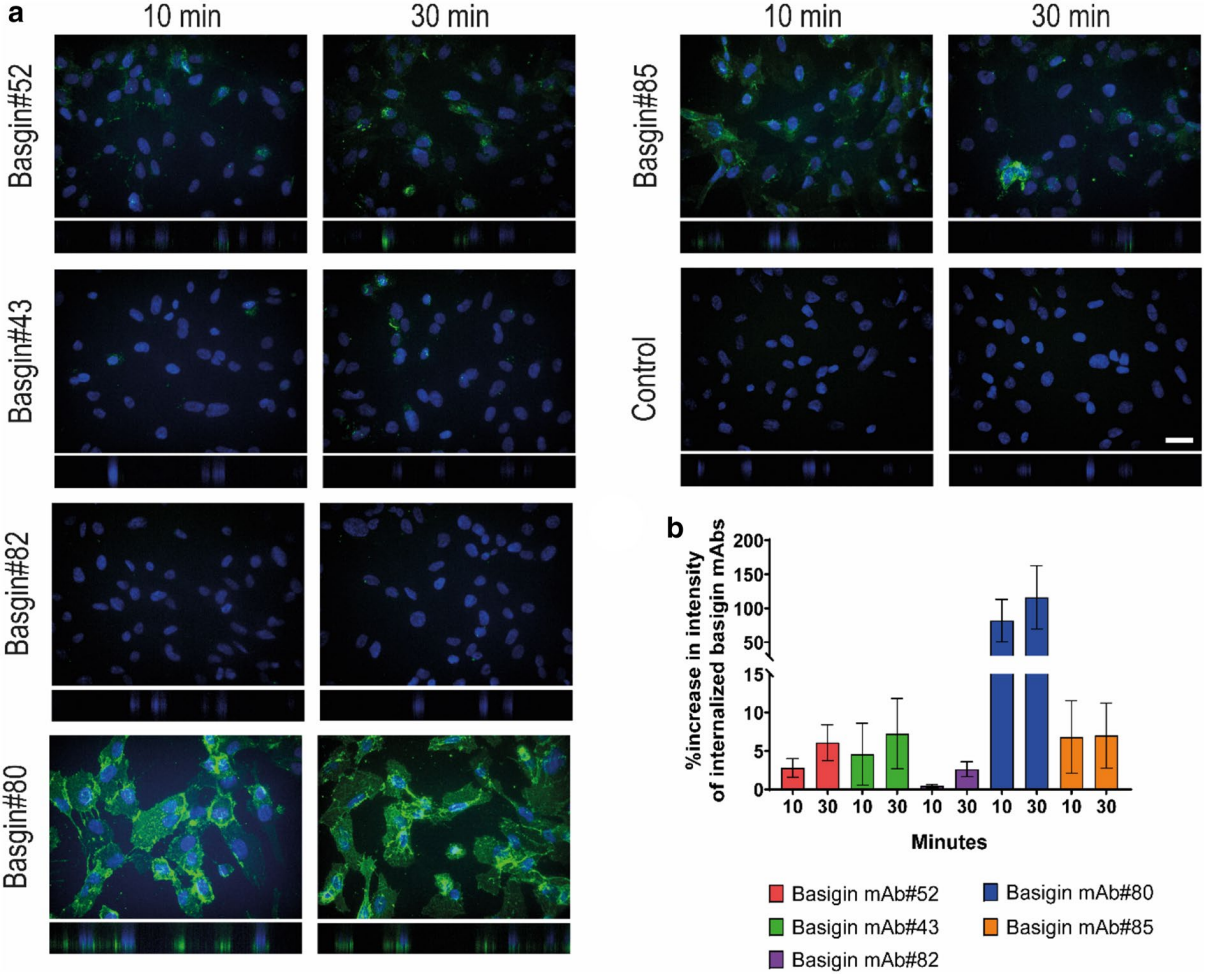
Internalization of basigin monoclonal antibodies (mAbs) in hCMEC/D3 cells. (**a**) Representative confocal images of selected basigin mAbs exposed to hCMEC/D3 cells for 10 and 30 min. Basigin mAbs in green and nuclei visualized by Hoechst in blue. The pictures are the maximum projection of the z-stack, and the XZ projection is below the pictures. Scale bar 30 µm. (**b**) Quantification of intracellular spots using Cellomics Arrayscan after acid stripping and staining. The intensities are normalized to the negative control and plotted as the percentage increase in spot intensity per cell with ± standard error of the mean (SEM).

Based on our results, five bin representative basigin antibodies, mAb#52 (bin A, K_D_ 3 nM), mAb#43 (bin B, K_D_ 20 nM), mAb#82 (bind C, K_D_ 20 nM), mAb#80 (bind D, K_D_ 20 nM), and mAb#85 (bin AD, K_D_ 0.4 nM), were selected and examined for their ability to support and trail receptor internalization in the hCMEC/D3 cells (Fig. 6). The antibodies were selected based on robust immunofluorescence staining of the hCMEC/D3 cells. The internalization was determined using high-content screening microscopy with 43 images per condition in three independent experiments and plotted as the percentage increase in spot intensity per cell compared to the control after acid-removal of surface-bound mAbs. The effect of the acid-removal was tested on surface stained cells with and without acid stripping, which resulted in a decrease in spot intensity in acid-treated, indicating that the spots quantified are mainly internalized mAbs (supplementary Figure S3). Additionally, representative confocal z-stack images were manually taken of the different time points (Fig. 6a). A time-dependent increase in basigin mAb internalization was observed for mAb#52, #43, #82, and #80, whereas high-affinity mAb#85 reached the same level of basigin-mediated internalization already at 10 min and did not change from 10 to 30 min (Fig. 6b). Basigin mAb#80 resulted in a much higher spot intensity compared to the other antibodies, which was not surprising since mAb#80 associated strongly with the hCMEC/D3 cells in the immunofluorescence staining (Fig. 5). However, the acid stripping was not efficiently removing mAb#80 from the cell surface, and some of the signal could, therefore, be from surface-bound mAbs. All the tested mAbs appear to allow for receptor internalization, making them relevant candidates as engineering partners to facilitate basigin-mediated transcytosis of therapeutic proteins across the BBB.

## Discussion

Zuchero et al.^1^ showed that targeting basigin increased the uptake of antibodies across the BBB in vivo. However, they did not provide details on their basigin mAb epitopes. Many studies have demonstrated that antibodies can be transported across the BBB, reaching the brain parenchyma using RMT^29^. However, in most cases, the antibodies used for these experiments are only selected based on their affinity and capability to bind the endogenous receptor in cells. In this study, we successfully generated a broad panel of mAbs targeting the basigin receptor. Here, the antibodies were selected by their epitope, which gave a broader panel of antibodies with possibly more different biological characteristics.

Based on binding to recombinant basigin-ECD by BLI, 21 mAbs out of the 54 were selected for further characterization. The mAbs were initially differentiated based on their epitope binding region, affinity, and ability to bind the basigin receptor on living cells. For comparison, the data for the 21 mAbs are summarized in Table 2. The alignment of the 21 mAb V_H_ sequences revealed nine CDR-H3 families, which were group into five epitope bins. Since it is known that affinity to the receptor can affect the transcytosis ability of the antibodies targeting the BBB^25,26^, the binding affinity of the selected 21 mAbs was determined.

**Table 2:** Summary of characterization of the basigin monoclonal antibodies.

mAb	V_H_ family	CDR-H3 family	Epitope bin	K_D_ (nM)	Flow cytometry	IF	Internalization
85	VH1	CDR-H3 8	AD	0.4	+	+ +	+ +
33	VH6	CDR-H3 3	A	0.3	+	+ +	
50	VH6	CDR-H3 3	A	1	+	+ +	
52	VH6	CDR-H3 2	A	3	+	+ + +	+ +
65	VH6	CDR-H3 2	A	4	+	+	
34	VH5	CDR-H3 1	B	7	+	+	
69	VH5	CDR-H3 1	B	9	+	+	
71	VH5	CDR-H3 1	B	10	+	+	
43	VH5	CDR-H3 1	B	20	+	+ + +	+ +
41	VH5	CDR-H3 1	B	20	+	+ + +	
59	VH5	CDR-H3 1	B	30	+	-	
42	VH5	CDR-H3 1	B	40	+	-	
81	VH6	CDR-H3 4	C	10	-	-	
82	VH1	CDR-H3 7	C	20	-	+ + +	( +)
83	VH6	CDR-H3 4	C	> 100	-		
74	VH1	CDR-H3 9	C	> 100	-		
73	VH1	CDR-H3 9	C	> 100	-		
76	VH1	CDR-H3 9	C	> 100	-		
75	VH1	CDR-H3 9	C	> 100	-		
79	VH6	CDR-H3 5	D	6	+	+	
80	VH3	CDR-H3 6	D	20	+	+ + +	+ + +
		21	21	21	21	16	5

*mAb* monoclonal antibody, *V*_*H*_* family* B-cell lineage, *CDR-H3 family* third complementarity-determining region of the heavy chain family, *K*_*D*_ Kinetic equilibrium dissociation constant, *IF* Immunofluorescence staining.

The flow cytometry analysis of the hCMEC/D3 cells showed that some basigin mAbs that did not associate with the endogenous receptor expressed in the hCMEC/D3 cells still bind basigin-ECD in the BLI analysis (Figs. 2 and 4). The BLI experiment and the flow cytometry analysis of the basigin mAbs allowed for both monovalent and bivalent binding to basigin. The bivalent interaction was dependent on the density of the basigin on the surface of the cells or biosensors, which could cause the differences between the positive binders in the epitope binning and flow cytometry analysis. The bin C mAbs did not bind the cells in the flow cytometry analysis at this concentration, and a higher concentration may be needed for cellular binding of the low-affinity bin C mAbs (K_D_ values > 100 nM).

Out of the 21 basigin mAbs, 16 were selected based on their epitope bin and affinity for the candidate selection step. The 16 mAbs represented all the epitope bins and had K_D_ values below 40 nM. They were tested for immunofluorescence staining of hCMEC/D3 cells to determine whether their epitope and/or affinity was important for their ability to detect the cellular basigin receptors. Overall, the differences in interaction with the basigin within each epitope bin were not explained by their differences in affinity. However, to thoroughly investigate the affinity dependency, affinity engineering of representative basigin mAbs would be needed to exclude the influence of individual and local epitopes. The basigin mAbs detection of the basigin receptor was also independent of their epitope, as an intense immunofluorescence staining was observed across the epitope bins. The contradictory results obtained with mAb#82 in the flow cytometry analysis and immunofluorescence staining could be explained by the fixation step, which could modulate the epitope making it more susceptible. In contrast, basigin mAb#42 did not reveal any immunofluorescence signal in the staining of cells, indicating that the antibody binds a conformational epitope, which cell fixation disrupts.

The basigin receptor exists in different glycosylated forms. It has been reported that the cellular localization of basigin is dependent on its glycosylation state. The lowly glycosylated basigin is suggested to be localized primarily in the endoplasmic reticulum, whereas the highly glycosylated basigin is proposed to be at the cell surface^22^. The glycosylation sites were conserved across species, indicating a functional or structurally important role of the glycan modifications^20^. Notably, the N-glycosylation is shown to be different between tissues and species^30^. The basigin receptors can form dimers, but whether the glycosylation affects the oligomerization is still debated. Glycosylation has been reported to enhance basigin receptor dimerization^21–23^, whereas other studies found that glycosylation was not essential^31,32^. The different glycosylated forms and dimerization patterns of basigin could explain the different abilities of the anti-basigin mAbs to detect membrane-associated basigin and their subcellular staining pattern. The extracellular domain of human basigin was subjected to enzymatic deglycosylation (Figure S4). The deglycosylation resulted in a shift in molecular weight shown by Western blotting. However, the deglycosylation did not affect the detection of human basigin using basigin mAb#52 (bin A) and mAb#80 (bin D) as these binds both the highly and lowly glycosylated forms.

Internalization is a crucial step in the selection process to identify mAbs capable of crossing the BBB. Five basigin mAbs, one representative for each epitope bin, were selected for internalization as part of the functional characterization. The internalization seemed to be increased from 10 to 30 min for all mAbs but mAb#85. The basigin-mediated internalization of the high-affinity mAb#85 did not change over time, indicating that mAb#85 has reached saturation already at 10 min. Basigin mAb #43, #82, and #80 had all K_D_ values of 20 nM, but their uptake levels were quite different. The difference could be explained by their epitope or receptor oligomer preference. mAb#80 resulted in a high internalization signal compared to the others, but the acid stripping of the hCMEC/D3 cell surface did not efficiently remove the mAb#80 surface binding, suggesting that mAb#80 were sticking to the cell surface. Also, the very strong interaction in the immunofluorescence staining could indicate that mAb#80 interacts with a specific subset of basigin receptors or that it exerts some unspecific interactions. The antibodies interact specifically with basigin shown by a substantial decrease in Western blotting band intensity after siRNA knockdown of basigin compared to samples were scramble siRNA was added (Figure S5). The subcellular localization of the internalized basigin mAbs was hard to interpret due to shrinkage of the cytoplasm after acid treatment.

We have generated a diverse panel of basigin mAbs binding to different epitope regions. In terms of the transcytosis capability of antibodies, a lot of the studies conclude only based on one epitope on a given receptor. It is important to explore the BBB crossing based on both affinity and epitope to be able to select lead candidates for drug delivery. Even though a previous study demonstrated that there was no correlation between epitope and function of basigin mAbs in T cells, this needs to be further analyzed in brain endothelial cells with our repertoire of antibodies^33^.

It remains to be confirmed if these basigin mAbs can transcytose with the receptor through the brain endothelial cells. The hCMEC/D3 cells are not optimal for testing transport across the cell layer due to the low tightness of the barrier model^34^. For such experiments, BBB in vitro models based on primary brain endothelial cells or human induced pluripotent stem cells are tighter and more suitable^35^. The basigin mAbs have cross-reactivity to at least two species (human and pig) out of the four tested (human, pig, rat, and mouse). The cross-reactivity eases test of the mAbs in preclinical models and progression of the lead candidates into clinical studies. The porcine cross-reactivity is convenient for in vitro modeling of the transcytosis using the robust porcine BBB model as well as a good human pharmacokinetic prediction using pigs for the in vivo experiments^36,37^.

Our work resulted in an excellent and broad panel of anti-basigin mAbs. If mAb#80 (bin D) is primarily binding the basigin receptor retained on the cell surface, it would not be relevant for basigin-mediated BBB delivery. Bin C mAbs cannot be excluded as possible candidates, but based on our studies, this concentration revealed weak cellular association and low uptake of mAb#82. In conclusion, bin A, B, and AD mAbs seemed to be promising candidates for further investigation based on their basigin-mediated internalization. The thorough characterization of the antibodies reported here provides valuable information for the further preclinical in vitro and in vivo characterization of the antibody repertoire and basigin-based BBB transportation.

## Methods

### Epitope binning using biolayer interferometry

The epitope binning assay was performed at Octet RED384 (Pall Fortebio) in 384-tilted well plates in a tandem setup. All samples were prepared fresh in 1 × PBS-P + buffer-10x (GE Healthcare Life Sciences, cat#88,995,084), which also was used as an assay buffer. First, the Dip and Read streptavidin (SA) biosensors for kinetics (Pall Fortebio, cat#18–5,019) were dipped in assay buffer for 200 s to record a baseline step. Next, 0.5 µg/ml of the biotin-tagged basigin-ECD was loaded on the biosensors for 200 s. After 200 s wash in buffer, the basigin-ECD-coated biosensors were dipped in 100 nM saturating basigin mAb (mAb1) for 1,200 s to reach saturation. The mAb1-basigin coated biosensors were washed for 200 s in buffer and then moved to wells containing an array of blocking basigin mAbs (mAb2) for 1,200 s. Complete self-blocking was ensured with all the basigin mAbs. Basigin mAbs with a baseline drift > 5% were only used as a blocking antibody. The data analysis was performed by using ForteBio Data Analysis software (version 10.0, Fortebio, Fremont, CA, USA), and epitope binning matrices were exported to Microsoft Excel. Data normalization was conducted by division of the mAb2 signal by the reference signal (mAb2 only) and multiplied with 100. The Pearson correlation coefficients were calculated by rows, and columns were ordered according to their correlation. The highest correlations defined as Pearson's correlation coefficient above 0.9 (red), weak correlation as between 0 and 0.9 (light red), and negative correlation below 0 (green).

### Surface plasmon resonance analysis of basigin monoclonal antibodies

Binding affinity analysis of basigin mAbs to basigin-ECD was performed on a Biacore S200 and T200 (GE Healthcare Life Sciences) using a kinetic capture setup on a CM4 chip. The CM4 surface was immobilized with a monoclonal mouse anti-human IgG (Fc) antibody (GE Healthcare Life Sciences, cat#BR100839) using amine coupling (GE Healthcare Life Sciences, cat#BR100050). The chip surface was initially activated with a 1:1 (v/v) mixture of 75 mg/ml 1-ethyl-3-(3-dimethylaminopropyl) carbodiimide hydrochloride (EDC) and 11.5 mg/ml N-hydroxysuccinimide (NHS). The anti-human IgG antibody was diluted in 10 mM sodium acetate to 25 µg/ml and immobilized at a flow rate of 10 µl/min for 7 min. The activated carboxylate groups were blocked by the injection of 1 M ethanolamine hydrochloride-NaOH, pH 8.5. During immobilization, HBS-EP + (GE Healthcare Life Sciences, cat#BR100188) was used as a running buffer. The basigin mAbs were adjusted to a concentration of 1 µg/ml in running buffer and captured on anti-human Fc mAb immobilized sensor followed by injection of basigin-ECD for 200 s and buffer for 300–600 s (association and dissociation, respectively) at a flow rate at 30 µl/min at concentrations ranging from 600–0 nM. No basigin antibody was captured in the reference flow channel (FC1). At the end of each cycle, the sensor surface was regenerated in 3 M MgCl_2_ for 30 s. 1 × HBS-P + (GE Healthcare Life Sciences, cat#BR-1003–68) with 1 mg/ml bovine serum albumin (BSA) (Rockland, cat#BSA-30) was used as running buffer for the kinetic analyses. Fitting of reference (FC1) subtracted data was performed using the Biacore S200 and T200 evaluation software (version 1.0, GE Healthcare Life Sciences, Uppsala, Sweden) using the pre-defined Langmuir 1:1 interaction model. The association rate constant (k_a_), dissociation rate constant (k_d_), and flow rate-independent component (tc) were fitted global, R_max_ fitted local, and bulk response (RI) fitted constant. For some of the kinetics analyses, the high concentration of basigin-ECD was excluded due to binding to the reference. The K_D_ values are based on two independent experiments.

### Flow cytometry analysis

The cultured hCMEC/D3 cells were detached with Versene (Gibco Life Technologies, cat#15,040–033), resuspended in flow cytometry buffer (phosphate-buffered saline (PBS), 2% normal goat serum (Jackson ImmunoResearch, cat#005–000-121), 2 mM EDTA), and plated in a 96 well round bottom plate with 1–0.5 × 10^6^ cells/well. Cells were stained with Live/dead fixable violet dead cell stain (Thermo Fisher Scientific, cat#L34955) for 15 min and washed twice in flow cytometry buffer before blocking in blocking buffer (PBS, 5% normal goat serum, 2 mM EDTA) for 10 min at 4 °C. The cells were incubated with 1 µg/ml primary basigin antibodies diluted in flow cytometry buffer for 30 min at 4 °C. After being washed twice in flow cytometry buffer, cells were incubated with 2.5 µg/ml secondary goat anti-human IgG Alexa Fluor 647 (Jackson ImmunoResearch, cat#109–605-008) for 30 min at 4 °C. The cells were fixed with BD Cytofix/CytoPerm (BD Biosciences, cat#554,714) for 15 min on ice. As negative controls for the staining procedure, cells were either incubated with secondary goat anti-human IgG Alexa Fluor 647, Live/dead fixable violet dead cell stain, or with buffer only (unstained). Since all the basigin mAbs are the same isotype, the basigin mAbs that did not bind the cells worked as Isotype controls. The samples were acquired using FACSVerse flow cytometry system (BD Biosciences, San Jose, CA, USA) with the BD FACSuite software calibrated with FACSuite CS&T research beads (BD Biosciences, cat#650,622), and analyzed using FlowJo (version 10, Tree Star, Inc., Ashland, OR, USA). The cells were gated based on the forward and side scatters to remove cell debris with an additional viability gate. Pulse geometry gating was used to remove doublets, and the final flow cytometry analysis was based on 50,000 events collected in the single-cell gate for each sample (Supplementary Figure S1). The setup was run three times, and the median fluorescence intensities were log-transformed and plotted in GraphPad Prism 8.0 (GraphPad Software, Inc, CA, USA). The fold change was back-transformed in order to describe the mean differences between samples.

### Internalization assay

hCMEC/D3 cells were seeded in 96 well plates (Perkin Elmer, cat#3,904) coated with rat tail collagen type 1 at a cell density of 15,000 cells per well. The cells were incubated with 1 µg/ml of the selected basigin mAbs for 10 and 30 min. The cell surface was acid stripped with 0.2 M acetic acid/0.5 M NaCl in PBS for 4 min on ice to remove surface-bound antibodies (as described in^38^) and subsequently washed in PBS and fixed in 4% PFA for 10 min at room temperature. After permeabilization with PBS 0.1% Triton X-100 (Sigma-Aldrich, cat#T9284) for 10 min, the internalized basigin antibodies were detected by 1 h incubation at room temperature with Alexa Fluor 568 conjugated goat anti-human secondary antibody (2 µg/ml) (Invitrogen, cat#A21090) diluted in PBS with 2% BSA. Nuclei were stained with Hoechst34580 (2 µg/ml) (Invitrogen, cat#H21486) diluted in H_2_O for 10 min. After being washed, the cells were analyzed in PBS. Cells only treated with secondary antibody were used as a negative control. All samples were in triplicates, and the experiment was performed three times. Quantification of basigin mAb internalization was done using Cellomics Arrayscan VTI setup (Thermo Fisher Scientific) equipped with an Orca-ER camera (Hamamatsu) using a Zeiss 20 × LD Plan-Neofluar (0.4 numerical aperture (NA)) objective and analyzed with the Spot Detector BioApplication. The algorithm was set to exclude dead cells based on nuclear size and fluorescence intensity. The intensity of the spots with fluorescence intensity greater than the pre-defined background level was quantified and normalized to the cell number per field. No region of interest was applied in this experiment. The three repeated experiments were normalized to the negative control and plotted as internalization signal above control levels in GraphPad Prism 8.0 (GraphPad Software, Inc, CA, USA).

### Confocal microscopy

Confocal images were acquired using UltraVIEW VoX Spinning Disk Confocal microscope (Perkin Elmer) with Nikon Eclipse Ti inverted microscope coupled to a CSU-X1 spinning disk head (Yokogawa) and equipped with a Nikon Ti 40x/0.95 NA air objective. Images were acquired with a c13440 Orca-flask 4.0 CMOS camera (Hamamatsu) with settings kept constant between samples unless other indicated. Additional confocal images were acquired with a Leica TCS SP8 confocal laser-scanning microscope (Leica Microsystems) using an HC PL APO CS 40x/0.85 NA air objective, and PMT and HyD detectors. At least three z-stack images were acquired per sample with a z-step size of 0.2 µm (Nikon microscope) or 0.6 µm (Leica microscope). Image processing was done with Volocity (version 6.4, Perkin Elmer, Waltham, MA, USA) and Fiji (NIH)^39^. Images were exported as raw TIFF files, and only adjustments to contrast and brightness were made. Pictures are shown as maximum XY projection of the entire Z-stack and slices of XZ cross-section. The size of scale bars is indicated in the figure legends.

### Statistical analyses

All the raw data or the log-transformed data were normally distributed shown by QQ plots. Multiple comparisons were performed by one-way ANOVA with Dunnett's multiple comparison post hoc test. Results are presented as mean ± standard error of the mean (SEM) with *p*-values < 0.05 considered as statistically significant, ***p value < 0.001. All the statistical analyses were performed using GraphPad Prism 8.0 (GraphPad Software, Inc, CA, USA).

## Supplementary information

Supplementary file1
