# Supplementary material for: Characterization of basigin monoclonal antibodies for receptor-mediated drug delivery to the brain
Source: Sci Rep. 2020 Sep 3;10:14582. doi: 10.1038/s41598-020-71286-2 (PMC7471916; doi:10.1038/s41598-020-71286-2)

Supplemental materials

**Characterization of basigin monoclonal antibodies for receptor-mediated drug delivery to the brain**

Sarah Christine Christensen ^1,2^, Berit Olsen Krogh ^2^, Allan Jensen ^2^, Christian Brix Folsted Andersen ^1^, Søren Christensen ^2^, Morten Schallburg Nielsen ^1*^

*^1^ Department of Biomedicine, Aarhus University, Aarhus, Denmark; ^2^ Department of Biotherapeutic Discovery, H. Lundbeck A/S, Copenhagen, Denmark*

Supplemental materials include:

- Additional methods
- Flow cytometry analysis gating strategy (Figure S1)
- Flow cytometry analysis histograms (Figure S2)
- Effect of acid stripping on surface staining of hCMEC/D3 cells (Figure S3)
- Binding to deglycosylated basigin (Figure S4)
- Specificity of the basigin antibodies (Figure S5)

**Additional methods**

*Cells*

The hCMEC/D3 cells were obtained under license (Millipore, cat#SCC066) and grown as described by the manufacturer^1^. Briefly, cells were grown in EndoGRO MV Complete Media kit (Millipore, cat#SCME004) supplemented with 1% Penicillin-Streptomycin (P/S) (Gibco, cat#15140122) and basic fibroblast growth factor (1 ng/ml) (Millipore, cat#GF003) under 5% CO2 at 37°C in rat tail collagen type I (0.25 mg/ml) (Millipore, cat#08-115) coated culture flasks, plates, or glass coverslips for immunofluorescence studies. Cells were used between passages 8 and 30 for experiments. The HEK293 cells (ATCC) were grown in DMEM supplemented with 10% fetal bovine serum and 1% P/S. The HEK293 6E cells were grown in F17 medium (Invitrogen, cat#A-1383501) supplemented with 0.1% Kolliphor P188 (Sigma-Aldrich, cat#K4894), 4 mM L-Glutamine (Invitrogen, cat#25030032), and 25μg/ml G418 (Invitrogen, cat#11811-031).

*Antibody generation*

The basigin-ECD sequence was cloned into a pTT5 vector^2^ and expressed transiently in HEK293 6E cells (cells and vectors were both licensed from Canadian National Research Council) using PEIPro as transfection reagent in a DNA-PEIPro ratio of 1:2. Two versions of basigin-ECD were produced with either a C- or N-terminal His-tag coupled to an enzymatically cleavable linker. The basigin-ECD proteins were purified using HisTrap column (GE Healthcare Life Sciences, cat#17-5248-02), and the fractions were analyzed by a Coomassie-stained SDS-PAGE. The protein purity and mass were analyzed by SDS-PAGE and on mass spectrometry (TOF MS ES+), respectively. The final protein concentration was determined by the Pierce BCA protein assay kit (Thermo Fisher Scientific, cat#23225). BALB/c mice were co-immunized with a mixture of human basigin-ECD, where the N- or C-terminal His-tags were cleaved off. Mice with high serum titer by human basigin indirect ELISA were selected and fused to SP2/0 mouse myeloma cells. The hybridoma selection was made in media containing HAT media supplement followed by two rounds of limiting dilution cloning. The clones were screened for binding to human basigin-ECD by ELISA and counter-screened against porcine, rat, and mouse basigin-ECD for cross-reactivity determination. The antibody clones were sequenced from total RNA preparations, and mAbs were recombinantly produced in a mouse-human chimeric format with a human IgG1/κ (G1m3) Fc using the HEK293 6E cells described above but with a proprietary vector (Akeso Biopharma). The antibodies were purified by standard protein A affinity chromatography. SEC-HPLC and SDS-PAGE evaluated the purity of the mAbs. The endotoxin content was <5 EU/mg.

*Sequence alignment of variable heavy chain sequences of the basigin monoclonal antibodies*

The phylogram was generated using CLC Main Workbench (version 8.0.1, Qiagen, Aarhus, Denmark) using a Multiple Sequence Comparison by Log-Expectation (MUSCLE) alignment of the heavy chain variable domain sequences with the Unweighted Pair Group Method with Arithmetic Mean (UPGMA) algorithm, Jukes-Cantor distance measure, and 10,000 bootstrap replicates^3^. Phylograms generated with CLC progressive alignment and ClustalW with UPGMA, Jukes-Cantor distance measure, and 10,000 bootstrap replicates were very similar to the MUSCLE generated^4^.

*Immunofluorescence staining*

hCMEC/D3 cells were seeded at 50,000 cells per Menzel Ø12mm #1.5 coverslip (Thermo Fisher Scientific, cat#11846933). The coverslips were coated with rat tail collagen type I. The cells were fixed in 4% paraformaldehyde (PFA) for 10 min at room temperature. The cells were washed three times in PBS and permeabilized with PBS 0.1% Triton X-100 (Sigma-Aldrich, cat#T9284) for 10 min. The cells were then blocked in PBS 2% BSA (Sigma-Aldrich, cat#A9418) followed by 1 h incubation at room temperature with basigin mAbs diluted to 1 µg/ml in PBS 2% BSA. After repeated washes, the cells were incubated with Alexa Fluor 568 conjugated goat anti-human secondary antibody (2 µg/ml) (Invitrogen, cat# A21090) for 1 h at room temperature. Nuclei were stained with Hoechst 34580 (2 µg/ml) (Invitrogen, cat#H21486) diluted in H_2_O for 10 min. After being washed, the coverslips were mounted on slides in fluorescence mounting medium (DAKO, cat#S3023).

*Western blotting of recombinant basigin*

Recombinant human basigin-ECD was treated with PNGaseF for 1 and 4 h at 37°C, according to the manufacturer (New England Biolabs, cat#P0704S). An equal amount of protein (5 μg/well) was mixed with 20 mM DTT and 1x NuPAGE LDS sample buffer (Invitrogen, cat#NP0007) and, after heated at 95°C for 5 min, loaded to NuPAGE 4-12% Tris-Bis SDS-PAGE (Invitrogen). The proteins were transferred to PVDF membranes by Novex iBlot Dry blotting system (Invitrogen, cat#IB24001) and blocked in Odyssey Blocking buffer/PBS (LI-COR Biosciences, cat#92740000) diluted 2x in PBS for 1 h at room temperature. The membranes were incubated overnight with primary basigin mAbs at 4°C (1 μg/ml). After extensive washing in PBS 0.1% Tween-20, secondary IRDye 800CW Goat anti-Human IgG Secondary Antibody (LI-COR Biosciences, cat#926-32232) and goat anti-mouse IgG (H+L) Highly Cross-Adsorbed Secondary Antibody, Alexa Fluor 680 (Invitrogen, cat#21058) (1:10,000) were applied for 1 h at room temperature. After washing, the fluorophore-emitted light was detected at 680nm and 800nm using Odyssey CLx near-infrared scanner (LI-COR Biosciences) with the same gain settings for all blots. Chameleon Duo Pre-stained Protein Ladder (LI-COR Biosciences, cat#928-60000) was used as a size reference.

*siRNA knockdown of basigin*

HEK293 cells were seeded in a 12well plate at a cell density of 200,000 cells/well in standard growth media 24 h prior to transfection. The HEK293 cells were transfected with 10pmol siRNA using Metafectene SI (Biontex, cat#T100-1.0) in Opti-MEM-GlutaMAX (Thermo Fisher Scientific, cat#51985026) without any additives. A pool of siRNA BSG1 (Sense 5’-GGUUCUUCGUGAGUUCCUCdTdT-3’, antisense 5’- GAGGAACUCACGAAGAACCdTdT-3’) and BSG2 (Sense 5’-GCUACACAUUGAGAACCUGdTdT-3’, antisense 5’- CAGGUUCUCAAUGUGUAGCdTdT-3’) were used. A non-targeting siRNA served as a negative control (Sense 5’-UUCUCCGAACGUGUCACGUdTdT-3’, antisense 5’-ACGUGACACGUUCGGAGAAdTdT-3’). All siRNAs were synthesized by GenePharma, Shanghai. The media were replaced with normal growth media after 4 h. 48 h after transfection, the cells were washed in PBS and harvest in 2x LDS buffer and boiled at 95°C for 10 min. The cell lysates were separated on an ExpressPlus 4-12% Bis-Tris PAGE (Genscript) using NuPAGE MES SDS Running Buffer (Invitrogen, cat#NP0002). The proteins were transferred to nitrocellulose membranes (Invitrogen, cat#IB23001) using Novex iBlot Dry blotting system (Invitrogen) and blocked in Tris-buffered saline (TBS) + 0.1% Tween 20 (TBST) with 5% skim milk powder for 1 h. The membranes were blotted with indicated basigin mAbs (1 μg/ml) and mouse anti-VTI1A (BD Transduction Laboratories, cat#611220, lot#5205919, immunogen mouse Vti1a aa.114-217) as a loading control (0.25 μg/ml) diluted in TBST 5% skim milk at 4°C overnight. The membranes were washed in TBST 1% skim milk and incubated with secondary antibodies (goat anti-human HRP-linked (Millipore, cat#AP112P) or horse anti-mouse HRP-linked (Cell Signaling, cat#7076s)) diluted 1:2000 in TBST 5% skim milk for 1 h at room temperature. After repeated washes in TBST 1% skim milk, the immunoreactive bands were visualized on the ImageQuant LAS 4000 (GE Healthcare Life Sciences) using Amersham ECL Prime Western Blotting Detection Reagent (GE Healthcare Life Sciences, cat#RPN2106) for development. SeeBlue Plus2 Pre-stained Protein Standard (Invitrogen, cat#LC5925) was used as a molecular weight reference. The bands were quantified using Fiji (NIH)^5^, and the intensities were normalized to the loading control. The results were plotted in GraphPad Prism 8.0 (GraphPad Software, Inc, CA, USA).

**References**

1. Weksler, B. B. *et al.* Blood-brain barrier-specific properties of a human adult brain endothelial cell line. *FASEB J.* **19**, 1872–1874 (2005).

2. Durocher, Y., Perret, S. & Kamen, A. High-level and high-throughput recombinant protein production by transient transfection of suspension-growing human 293-EBNA1 cells. *Nucleic Acids Res.* **30**, E9 (2002).

3. Edgar, R. C. MUSCLE: multiple sequence alignment with high accuracy and high throughput. *Nucleic Acids Res.* **32**, 1792–7 (2004).

4. Thompson, J. D., Higgins, D. G. & Gibson, T. J. CLUSTAL W: improving the sensitivity of progressive multiple sequence alignment through sequence weighting, position-specific gap penalties and weight matrix choice. *Nucleic Acids Res.* **22**, 4673–80 (1994).

5. Schindelin, J. *et al.* Fiji: an open-source platform for biological-image analysis. *Nat. Methods* **9**, 676–682 (2012).

**Figure legends**

**Figure S1.** Gating strategy for flow cytometry analysis.

**Figure S2.** Histograms of flow cytometry analysis. Histograms in red depict secondary antibody control, and blue histograms the basigin signal detected with different basigin monoclonal antibodies (mAbs) indicated above the histograms.

**Figure S3.** Basigin surface staining for 90 min 4°C. The hCMEC/D3 cells were treated with and without 0.2 M acetic acid/0.5 M NaCl in PBS for 4 minutes on ice. The fluorescence signal of spots/cell was plotted with ± standard error of the mean (SEM).

**Figure S4.** The specificity of selected basigin monoclonal antibodies (mAbs) by siRNA knockdown of basigin in HEK293 cells. **a** Western blot of siRNA knockdown of basigin. **b** Quantification of the intensity of the bands in the Western blot normalized to the loading control VTI1A (Vesicle Transport Through Interaction With T-SNAREs 1A). NC: negative control with a non-specific siRNA, BSG pool: a pool of two different basigin siRNAs.

**Figure S5.** The specificity of selected basigin monoclonal antibodies (mAbs) by siRNA knockdown of basigin in HEK293 cells. **a** Western blot of siRNA knockdown of basigin. **b** Quantification of the intensity of the bands in the Western blot normalized to the loading control VTI1A (Vesicle Transport Through Interaction With T-SNAREs 1A). NC: negative control with a non-specific siRNA, BSG pool: a pool of two different basigin siRNAs.

Figure S1

Figure S2

Figure S3


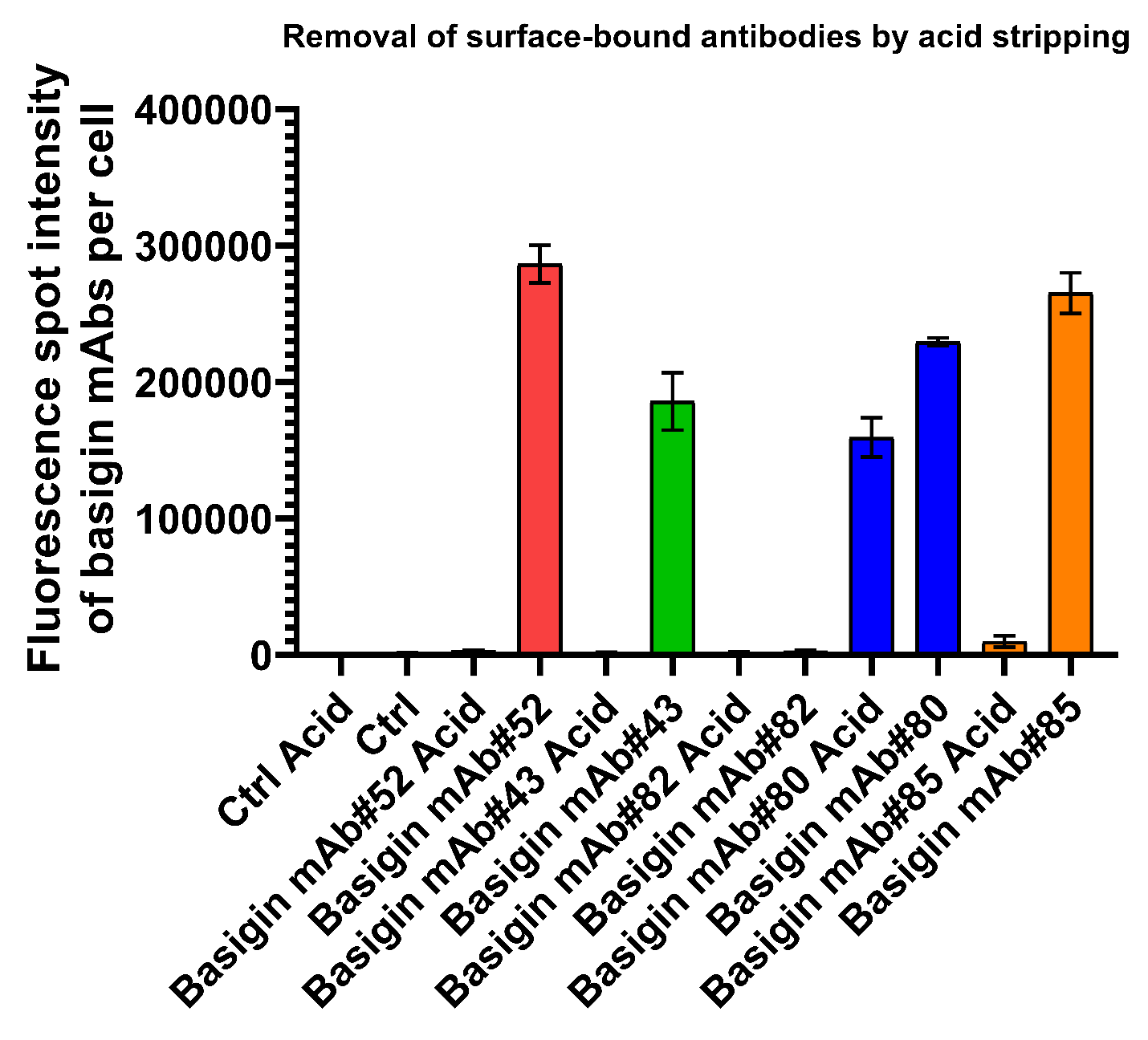


Figure S4


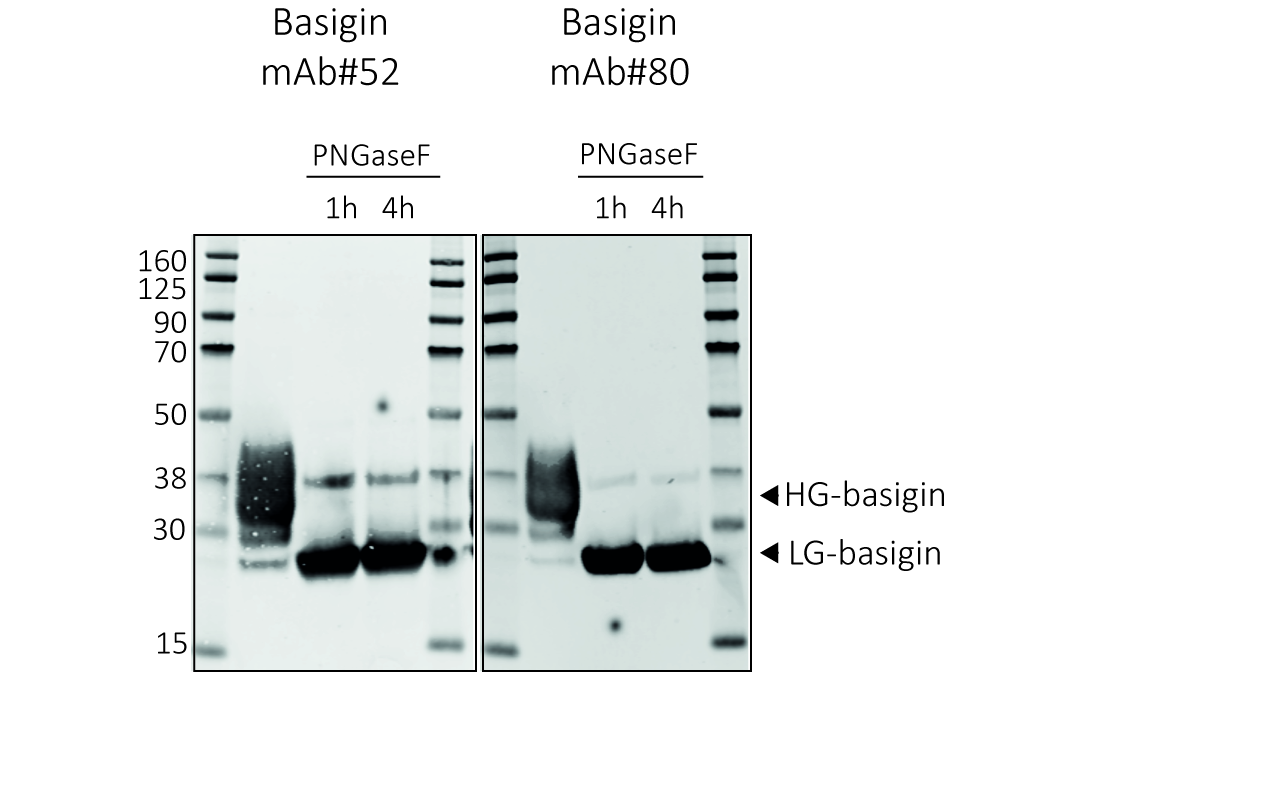


Figure S5


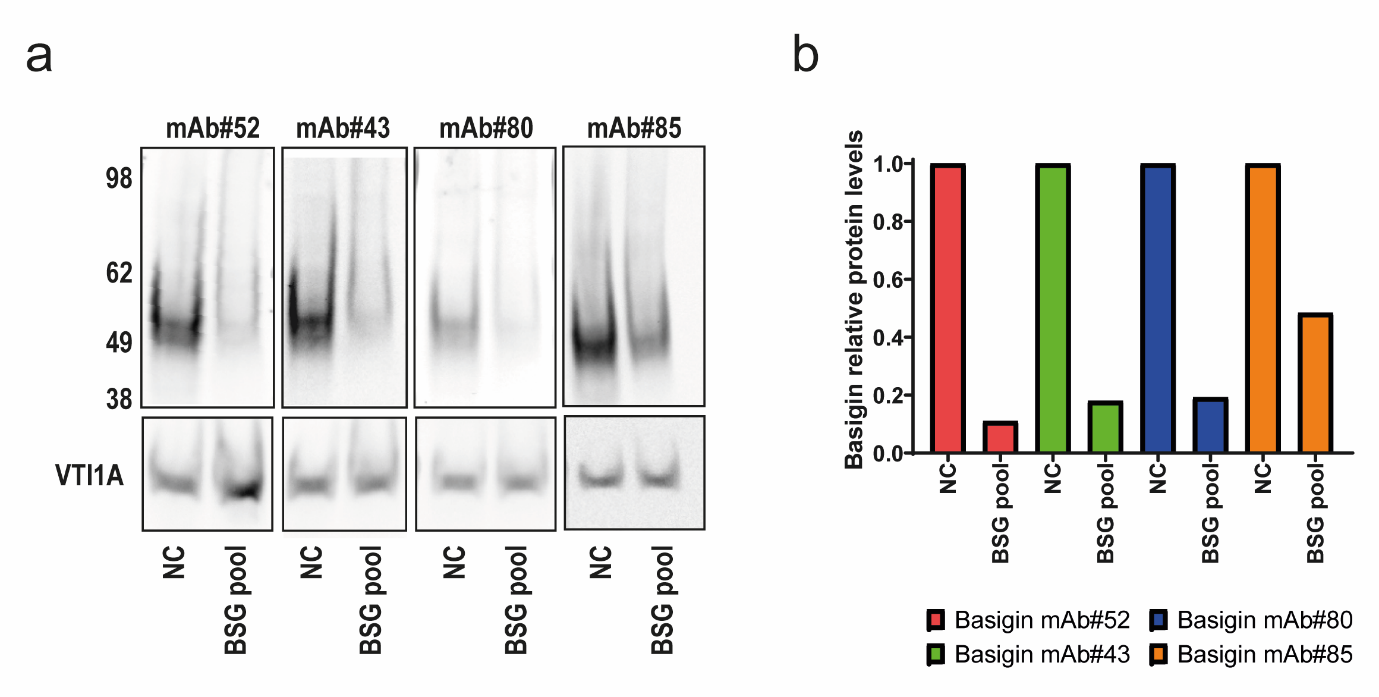

Supplement: Supplementary file 1 — Supplementary file1 [file 41598_2020_71286_MOESM1_ESM.docx]
